# A Parallel Adaboost-Backpropagation Neural Network for Massive Image Dataset Classification

**DOI:** 10.1038/srep38201

**Published:** 2016-12-01

**Authors:** Jianfang Cao, Lichao Chen, Min Wang, Hao Shi, Yun Tian

**Affiliations:** 1 Department of Computer Science & Technology, Xinzhou Teachers University, Xinzhou 034000, China; 2 School of Computer Science & Technology, Taiyuan University of Science and Technology, Taiyuan 030024, China

## Abstract

Image classification uses computers to simulate human understanding and cognition of images by automatically categorizing images. This study proposes a faster image classification approach that parallelizes the traditional Adaboost-Backpropagation (BP) neural network using the MapReduce parallel programming model. First, we construct a strong classifier by assembling the outputs of 15 BP neural networks (which are individually regarded as weak classifiers) based on the Adaboost algorithm. Second, we design Map and Reduce tasks for both the parallel Adaboost-BP neural network and the feature extraction algorithm. Finally, we establish an automated classification model by building a Hadoop cluster. We use the Pascal VOC2007 and Caltech256 datasets to train and test the classification model. The results are superior to those obtained using traditional Adaboost-BP neural network or parallel BP neural network approaches. Our approach increased the average classification accuracy rate by approximately 14.5% and 26.0% compared to the traditional Adaboost-BP neural network and parallel BP neural network, respectively. Furthermore, the proposed approach requires less computation time and scales very well as evaluated by speedup, sizeup and scaleup. The proposed approach may provide a foundation for automated large-scale image classification and demonstrates practical value.

Image classification uses automated analysis to categorize images^1^. Image classification has been widely applied for target detection and recognition, image retrieval, information filtering and so on in fields such as information search, security monitoring, medical information, and aerospace. Therefore, effectively and accurately dividing images into appropriate categories has been the focus of much research.

Studies on image classification technologies have concentrated primarily on classification approaches. Based on the design concept of the classifiers, image classification can be divided into two approaches: the classification approach based on the generative model and the classification approach based on the discriminant model. The classification approach based on the generative model is based on the joint probability distribution of image features and image categories. The simplest approach to image classification based on the generative model is the Bayesian model. Ducinskas *et al*. presented Bayes linear discriminant functions and applied them to image classification^2^. To improve image retrieval, Gao *et al*. used a Simple Bayesian Classifier to perform semantic classification of image resources^3^. The most common approach for image classification based on the generative model is the Gauss Mixture Model (GMM). Valiaya *et al*. proposed a hierarchical image classification method through modeling image features. They first performed classification on different levels for a vacation image dataset and then practiced Gauss mixture modeling for each class to achieve the whole vacation image classification^4^. With the wide application of the bag-of-words model in the image fields, various types of topic models have become widely used for image classification. One of the most common models is Probabilistic Latent Semantic Analysis (PLSA), which represents an image as a set of visual words using the bag-of-words model, regards the image category as a potential theme, and then finds the distribution of latent semantics by analysis^5^. The PLSA model was first proposed for document processing aiming at discrete text information. To better adapt this model to image data, Horster *et al*. proposed an approach of modeling the distribution of visual word elements using the distribution of continuous local feature vectors. Experiments showed that this method was superior to the traditional discrete visual word element model^6^. Because the image classification approach based on the generative model expresses the data distribution from the perspective of statistics and can reflect the similarity of similar data, its expansion capability is stronger and can produce good results in the case of more sample data or incomplete data information. However, the model directly determines the complexity of the algorithm. The complexity of a simple model is small, but with fewer parameters, the descriptive ability is poor; the descriptive ability of complex models is strong, but the algorithm complexity is high due to the inclusion of more parameters^7^.

Image classification approaches based on the discriminant model are established on the basis of the conditional probability distribution of the image features and the image categories. Artificial Neural Networks (ANN), decision trees and Support Vector Machines (SVM) are commonly used image classification approaches based on the discriminant model. ANN simulates the working process of biological neural networks to realize image classification, and strong adaptive learning ability is its most important feature. Kuruvilla *et al*. used neural network algorithms to classify computed tomography (CT) lung cancer images^8^. Han *et al*. proposed a neural network ensemble algorithm to classify remote sensing images^9^. To improve the classification accuracy rate, they employed radial basis function (RBF) neural networks as base classifiers and constructed a new classifier using the Rotation Forest algorithm. To obtain high discrimination of image representations from small datasets, Shi *et al*. proposed an image classification method based on a mixed deep transfer learning model^10^. Decision trees are an image classification approach based on inductive learning, which can deduce classification rules such as decision trees from data samples without order and rule. With the goal of classifying SAR images, Topouzelis *et al*. proposed a novel oil spill feature selection and classification technique based on a decision tree forest that constructed the optimum forest containing 70 trees^11^. Dou *et al*. studied decision tree classification technology and performed remote sensing image classification using the C5.0 algorithm, which improved both classification accuracy and effectiveness compared with a support vector machine (SVM) classifier^12^. SVM is one of the most widely used classification approaches in the image field. Liang *et al*. proposed a novel classification approach for hyperspectral remote sensing images based on the ICA and SVM algorithms^13^. This approach used the independent component analysis (ICA) algorithm to extract characteristic information from the hyperspectral remote sensing images. It then used the SVM algorithm to construct a classifier. To improve SVM classification performance, Pasolli *et al*. presented a new active learning approach that integrated spatial information from remote sensing images^14^. Image classification approaches based on the discriminant model can reflect the differences between data categories and determine the optimal classification surface between different data categories. The classification boundary is more flexible, which results in strong generalization ability and simple models that are easy to learn. Although it can solve complex nonlinear mapping problems, the artificial neural network has several disadvantages, such as overfitting, falling easily into a local optimum, and slow convergence speed. The classification accuracy of the decision tree algorithm drops dramatically when processing multiclass problems; although Bayes’ algorithm can make judgments along scientific lines, the analysis and calculation process is more complex. Moreover, some data must also use a subjective probability. The support vector machine has good generalization ability and can solve nonlinear high-dimensional problems, but it is difficult to determine its nuclear and penalty parameters, complicating the task of improving the classification model in practical applications^15^.

Subsequently, scholars proposed a number of optimization algorithms that improved image classification accuracy by optimizing the parameters of the traditional algorithms. Li *et al*. proposed a hybrid feature selection strategy to realize hyperspectral image classification by combining the genetic algorithm (GA) and SVM^16^. To solve the BP neural network’s problem of falling easily into a local minimum, Song *et al*. used the GA algorithm to optimize the initial parameters of BP neural network and then classified ETM+ remote sensing images^17^. Aiming at classifying fully polarimetric SAR images, Yu *et al*. presented a new supervised classification algorithm based on the BP neural network optimized by the particle swarm optimization (PSO) algorithm^18^. Xue *et al*. used the PSO algorithm to optimize the penalty parameter, C, and the kernel parameter, gamma, for the SVM classifier and constructed an optimized classification model for hyperspectral images^19^. Wang *et al*. introduced the ant colony optimization algorithm for image classification^20^. However, these optimization algorithms have some deficiencies. The search speed of GA is slow, and it is not capable of a good local search. When applied to discrete optimization problems, the PSO algorithm’s effect is not good, and it falls easily into local optimums. The time complexity of the ant colony algorithm is high, and its computational cost is large^21^.

In the era of big data, the number of available images has increased by orders of magnitude: from GBs to TBs or even PBs per day. In such an environment, the time efficiency and classification accuracy of traditional classification algorithms decline sharply, leading to new challenges. MapReduce is a parallel programming model used on the Hadoop platform^22^. Using this model, distributed parallel processing can be achieved without increasing the hardware costs involved, making it cost-effective to apply parallel processing to the traditional classification algorithms. At present, studies on the MapReduce model are in full swing. To obtain better generalization performance, He *et al*. proposed an efficient parallel extreme learning machine (ELM) for regression based on the MapReduce framework^23^. To enable large-scale distributed computing across multiple clusters, Wang *et al*. designed and implemented a MapReduce framework called G-Hadoop^24^. Ahmad *et al*. solved the problem of communications overlap in the MapReduce model by including Reduce in the overlap^25^. To improve reduction efficiency, Qian *et al*. proposed a parallel attribute reduction algorithm based on MapReduce^26^. Idris *et al*. proposed the MapReducePack, which executed a set of related algorithms in a single MapReduce job and, thus, extended MapReduce, which previously supported execution of only a single algorithm in the entire Hadoop cluster^27^. Matsuzaki *et al*. adopted parallel tree accumulations on MapReduce as the target computational pattern^28^. Zhang *et al*. proposed a BP neural network algorithm based on MapReduce and constructed a bank risk prediction model^29^. Although numerous studies on MapReduce exist, only a few studies address image classification directly using the MapReduce model.

In view of the problems described above, this paper proposes a new parallel Adaboost-BP neural network algorithm for classifying massive image datasets. This algorithm is a parallelized version of the traditional Adaboost-BP neural network algorithm that takes full advantage of the MapReduce parallel programming model and establishes a model for mass image classification. This study effectively improves the time performance and accuracy of image classification by employing distributed parallel processing techniques.

## Results and Discussion

**Figure 1:**
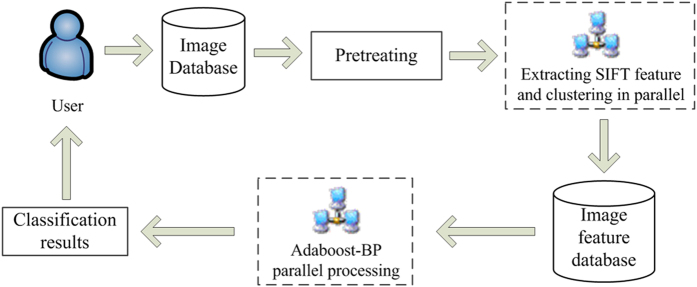
The classification model for massive image datasets.

**Figure 2:**
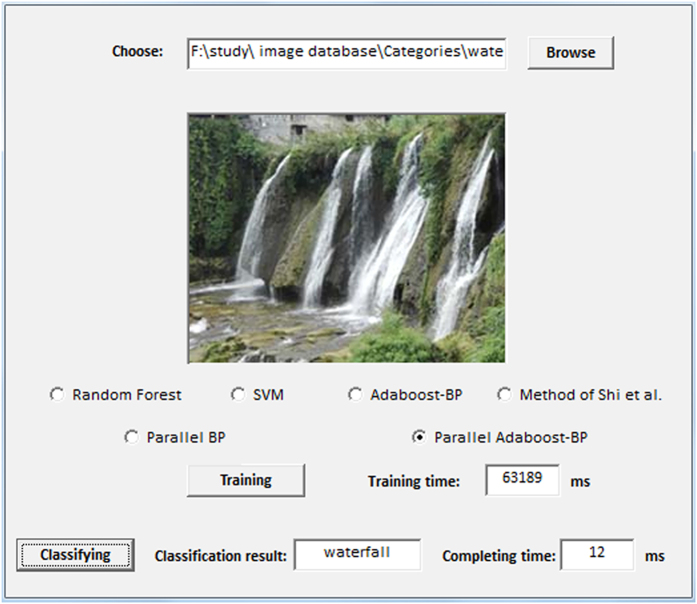
A snapshot of the automated image classification system user interface.

To evaluate the efficiency of the proposed approach, we implemented a prototype image classification system using the Java programming language. The automated system classifies images into the classes defined by the Pascal VOC2007, Caltech256 or SUN datasets according to the classification model shown in Fig. 1. The user interface of the prototype automated image classification system is presented in Fig. 2.

In addition, to validate the performance of the proposed algorithm, we conducted experimental comparisons by evaluating its classification accuracy, running time, speedup, sizeup, and scaleup.

### Classification accuracy

**Figure 3:**
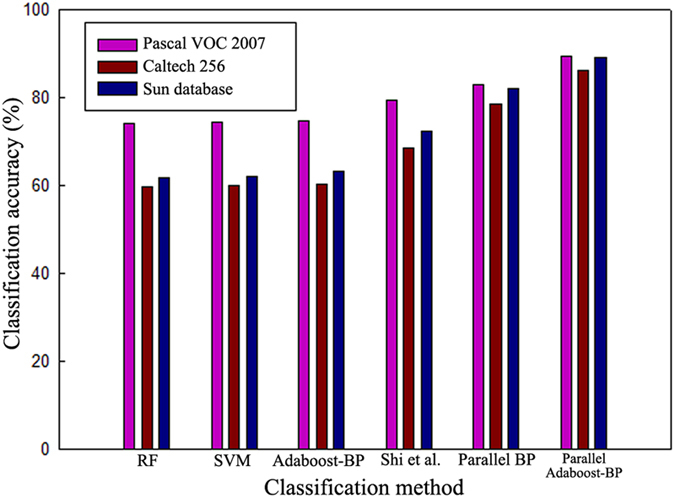
Comparison of the average accuracy (%) of the different approaches based on different datasets.

Based on the Pascal VOC2007, Caltech256 and SUN datasets, the traditional Random Forest (RF), Support Vector Machine (SVM), Adaboost-BP neural network algorithm^30^, the method of Shi *et al*.^10^, the parallel BP neural network algorithm^29^, and the parallel Adaboost-BP neural network algorithm proposed in this study were compared in terms of their classification precision ratios. The experimental results are shown in Tables 1 and 2 and in Fig. 3. Because Pascal VOC2007 has only 20 categories, whereas the Caltech256 and SUN datasets have many categories, the following lists the classification accuracy of all categories of the Pascal VOC2007 dataset. The classification accuracy of the other two datasets cannot be presented for all categories; thus, only the minimum, maximum and average values of the classification accuracy are given.

Table 1 shows a comparison of the classification accuracies of the tested classification approaches based on the 20 categories from the Pascal VOC2007 dataset. As Table 1 shows, the classification performance of the approach proposed in this study is more accurate than those of the other methods. Compared with the traditional RF, SVM, Adaboost-BP neural network algorithm and the method of Shi *et al*., the proposed approach achieved accuracy improvements of 15.1%, 15.0%, 14.5% and 10.0%, respectively. Compared with the parallel BP neural network algorithm, the classification accuracy improved by 6.4%. Although all six methods are based on the discriminant model, the classification accuracy of the traditional RF, SVM, Adaboost-BP neural network algorithm and the method of Shi *et al*. based on stand-alone architecture are significantly lower; however, that of the parallel BP neural network algorithm and the proposed approach based on the MapReduce parallel programming model are obviously higher. Moreover, the classification accuracy of the proposed approach is higher than that reported for the parallel BP neural network algorithm in due to the combination of the outputs of 15 BP neural networks that construct a strong classifier^29^. Consequently, the proposed approach leads to the lowest standard deviation, showing that fluctuations in the sample data affect it the least; therefore, it achieves the best classification results in the experiments.

Table 2 shows a comparison of the average classification accuracies of the tested classification approaches based on approximately 30,000 images from the Caltech256 and SUN datasets. Figure 3 shows the sharp contrast in average classification accuracy of different methods on the three datasets. In the case of the Caltech256 dataset, compared with the results in Table 1, the average classification accuracy of the traditional RF, SVM, Adaboost-BP neural network algorithm and the method of Shi *et al*. decreases by 14.5%, 14.3%, 14.6% and 10.7%, respectively. For the SUN dataset, the average classification accuracy decreases by 12.7%, 12.3%, 11.5% and 6.9%, respectively, but the accuracy of the parallel BP neural network algorithm and the proposed approach decreases by only 4.3% and 3.1%, 0.9% and 0.2% in case of Caltech256 dataset and SUN dataset, respectively, further indicating that the classification performance of the traditional algorithms based on stand-alone architecture will continue to decline as the amount of data increases. In contrast, the classification performance of the proposed algorithm based on the MapReduce model decreases only slightly because they use an applied distributed parallel processing architecture, in which greater amounts of data are simply processed using more of the computational capability of the clustered nodes.

**Figure 4:**
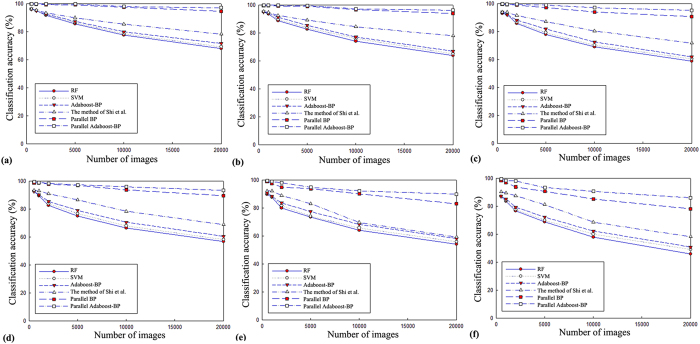
(**a**) The accuracy decline curve of different approaches with 5 images in each category. **(b)** The accuracy decline curve of different approaches with 10 images in each category. **(c)** The accuracy decline curve of different approaches with 20 images in each category. **(d)** The accuracy decline curve of different approaches with 50 images in each category. **(e)** The accuracy decline curve of different approaches with 100 images in each category. **(f)** The accuracy decline curve of different approaches with 200 images in each category.

In addition, to better verify the classification performance, we randomly selected images from different image categories (5, 10, 20, 50, 100, and 200 images per category) and total numbers of images (500, 1,000, 2,000, 5,000, 10,000, and 20,000—65% for training and 35% for testing) from the Pascal VOC2007, Caltech256 and SUN datasets for comparison purposes. The experimental results are shown in Tables 3 and 4 and Fig. 4.

Table 3 shows the average accuracies of the tested approaches based on different numbers of images and categories. Typically, for different classification algorithms, changing the proportions of the training set and test set would affect the classification accuracy for the same dataset. Increasing the number of the training set would improve the classification accuracy. Presumably, with the increase in the number of images and categories, the number of datasets may increase accordingly. As a consequence, the training and test processes will become more complex, which leads to a decrease in the classification accuracy. As Table 3 shows, regardless of which classification approach is used, when the number of images is fixed, classification accuracy decreases as the number of image categories increases. Similarly, when the number of categories is fixed, classification accuracy also decreases as the number of images increases. This result suggests that larger numbers of images and categories complicate the classification process, leading to declines in classification accuracy.

Table 4 lists the degree of decline of the classification accuracy of the different classification approaches with different numbers of images as the number of images in each category increases from 5 to 200. As Table 4 shows, increasing the number of categories has little effect on classification accuracy (except for the traditional RF, SVM and Adaboost-BP neural network algorithms) when there are fewer than 5,000 images. However, after the number of images surpasses 5,000, increasing the number of image categories has an obvious effect on classification accuracy, particularly on the algorithms based on single node architecture such as the traditional RF, SVM, Adaboost-BP neural network algorithm and the method of Shi *et al*.^10^. Therefore, as the number of images increases, the classification accuracy declines in the parallel algorithms (those based on parallel distributed processing architecture technology such as the parallel BP neural network algorithm and the proposed algorithm) are far smaller as the number of image categories increases. This finding is particularly true for the parallel Adaboost-BP algorithm proposed in this study, which is mainly due to construction of a strong classifier, although the classification accuracy of all the classification approaches declines.

Figure 4 (a–f) shows the declines in classification accuracy for the different classification approaches as the number of images and image categories increases. It shows that the traditional RF, SVM and Adaboost-BP neural network algorithms achieve the lowest classification accuracy, while the approach proposed in this study achieves the highest classification accuracy. Moreover, the classification accuracies of the different approaches are almost the same with fewer than 2,000 images, but they increasingly diverge in classification accuracy (particularly the traditional RF, SVM, Adaboost-BP neural network algorithm and the method of Shi *et al*.^10^.) as the number of images surpasses 2,000. In addition, as the number of image categories increases, the decline curves of classification accuracy for the two parallel algorithms are gentle as the number of images increases. This result indicates that increasing the number of images has little influence on the classification performance of parallel algorithms based on MapReduce and further illustrates the advantages of distributed parallel processing, which are even more apparent in the approach proposed in this study.

**Figure 5:**
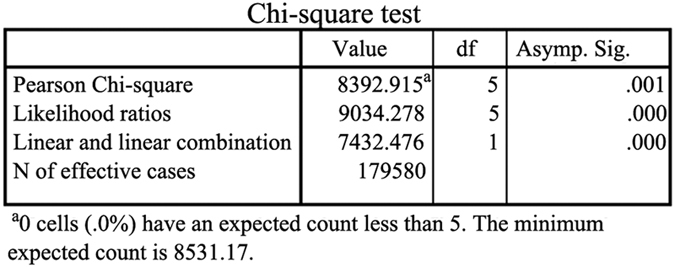
The results of the chi-square test.

Furthermore, to evaluate the statistical differences between the different approaches, we counted the number of correctly classified images and incorrectly classified images in the test sets of the three datasets according to the experimental results in Tables 1 and 2 (see Table 5) and then verified the reliability of the experimental results using the chi-square test for statistical analysis. Figure 5 lists the statistical analysis results using the chi-square method.

The data in Table 5 show that the performance of the traditional classification algorithms (such as RF, SVM, Adaboost-BP, and the method of Shi *et al*.) based on single node architecture is far worse than the performance of the parallel algorithms (such as parallel BP, the proposed approach in this study) based on MapReduce when used with a large number of datasets. In general, when using the chi-square test, if the value of Asymp.Sig is less than 0.05 and the number of cells of theoretical frequency less than 5 is not more than 20%, the experimental results are considered reliable. As shown in Fig. 5, the value of Asymp.Sig is 0.001 and 0 cells (.0%) have an expected count less than 5, which fully shows that the classification accuracy results obtained using different classification approaches are reliable.

### Running time

To further verify the effectiveness of the proposed approach, Table 6 reports the training and testing times for the different classification approaches while varying the number of images.

Table 6(a) shows the training time required for different numbers of images. The training time required by the RF, SVM, Adaboost-BP neural network algorithm and the method of Shi *et al*. are much longer than those of the two parallel algorithms because the two parallel algorithms adopt distributed parallel processing technology, while RF, SVM, Adaboost-BP neural network algorithm and the method of Shi *et al*. use single node architecture with limited processing capacity. The value in Table 6(b) shows the average testing time per image, which is calculated by dividing the total testing time by the total number of images tested. Again, the testing time required by RF, SVM, Adaboost-BP neural network algorithm and the method of Shi *et al*. is greater than that of the two parallel algorithms. In addition, the training and testing times of the parallel Adaboost-BP neural network algorithm proposed in this study are a little shorter than those of the parallel BP neural network algorithm. This difference mainly occurs because the proposed approach constructs a strong classifier using the Adaboost algorithm and employs the designed combine() function to process the intermediate results of the Map task locally. These decisions made during the process of parallel algorithm design effectively reduce the communication overhead among the nodes in the cluster.

### Speedup, sizeup and scaleup

For the parallel programming model based on MapReduce, we evaluate the performance of the proposed algorithm in terms of speedup, sizeup and scaleup^31^.

**Figure 6:**
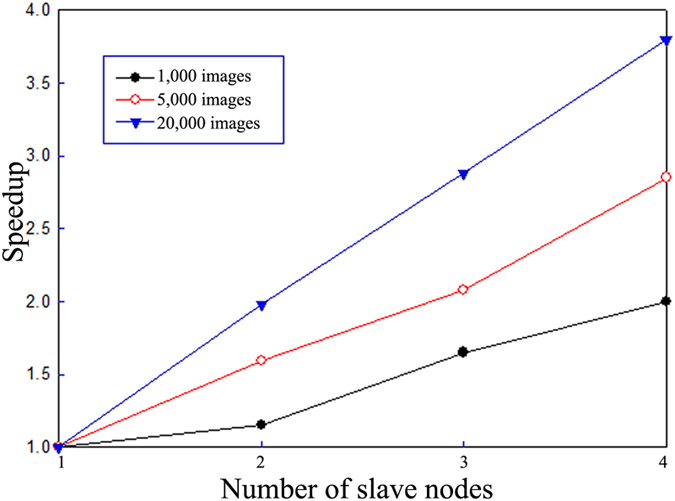
Speedup comparison.

Speedup refers to the ratio of the time required to run a task on a single calculating node to the time required to run that same task on multiple calculating nodes. To measure the speedup, we fix the size of the dataset and increase the number of node computers employed (e.g., from one node to four nodes). In addition, we investigated different numbers of images: 1,000, 5,000, and 20,000. The results are shown in Fig. 6.

In ideal conditions, speedup would grow linearly as the number of calculating nodes increases. However, because conditions can be affected by load balancing, communication overhead and other factors, speedup does not increase linearly. We find that the parallel Adaboost-BP neural network algorithm proposed in this study has good speedup performance. The maximal value of the speedup can reach approximately 3.8 when using four slave node computers. Moreover, as the number of images increases, the speedup increases as well. This primarily occurs because the time to process small numbers of images is not significant compared to the time consumed by communication and task arrangement; however, the intensive image computation becomes dominant as the number of images increases, and the speedup thus increases as well. Most importantly, as the number of image surpasses 20,000, the speedup is almost linear. Therefore, the proposed algorithm is appropriate for processing large datasets efficiently.

**Figure 7:**
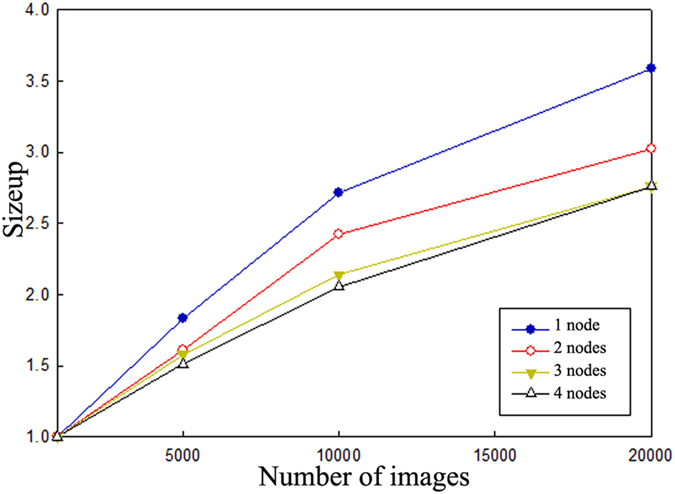
Sizeup comparison.

Sizeup is defined as the additional time required on a given system as the number of images becomes *m*-times larger than the original number of images. In other words, the greater the number of images, the higher the value of sizeup and the longer the cluster system would require to process them. To measure sizeup, we fix the number of the slave nodes to 1, 2, 3, or 4 while increasing the number of images from 1,000 to 20,000 images for each node. Figure 7 shows the experimental results. It is easy to see that when we increase the number of images from 1,000 to 20,000, the sizeup of the 1-node system increases by approximately 2.5, while it increases by only 1.5 for the 4-node system because the communication time of the 1 node system is smaller than that of the 4 node system, but the communication time does not increase much in the proposed parallel Adaboost-BP neural network algorithm as the number of images increases. Therefore, the proposed algorithm in this study has a good sizeup performance.

**Figure 8:**
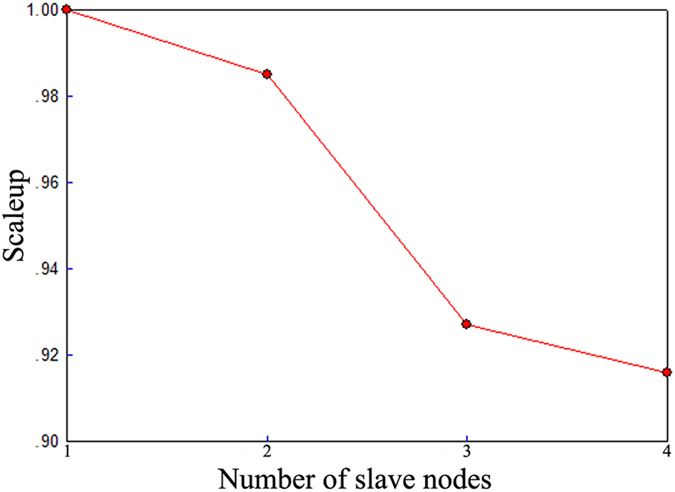
Scaleup comparison.

Scaleup refers to the ability of a system *m*-times larger to perform a job *m*-times larger in the same running time as the original system. The scaleup measure is used to evaluate the ability of an algorithm to adapt to both system growth and to increases in the number of images. Obviously, a higher scaleup value indicates better algorithm performance. Therefore, scaleup validates how well the algorithm handles larger numbers of images when more node computers are available. To evaluate the scaleup measure, we increase the number of node computers and the number of images simultaneously and obtain the scaleup values for the following combinations (1 node computer, 1,000 images), (2 node computers, 5,000 images), (3 node computers, 10,000 images) and (4 node computers, 20,000 images). The results are shown in Fig. 8. The higher the scaleup value, the better the performance. As Fig. 8 shows, the scaleup values are all higher than 0.90, demonstrating that the proposed algorithm scales well.

## Conclusions

Image classification is a complicated and time-consuming process, requiring space and time to select, extract, identify features and establish a classification model. The rapid development in information network technology today means that increasing numbers of images are available, far exceeding the abilities of traditional classification algorithms to use these images efficiently unless their calculation times drop dramatically. The open-source and distributed computing Hadoop platform has been widely adopted because it is both convenient and inexpensive to establish Hadoop clusters, and the platform has a simple and easy-to-use computing model. The academic and industrial worlds are continuously studying ways to adapt the traditional algorithms and applications developed for single machine or mainframe environments to the Hadoop cluster environment.

This study proposed a parallel Adaboost-BP neural network algorithm based on MapReduce for massive image dataset classifications. Moreover, it conducted an in-depth exploration and analysis of the parallel design and implementation of the Adaboost-BP neural network algorithm. The following three topics were investigated: construction of a strong classifier using the Adaboost algorithm, the parallel design and implementation of the Adaboost-BP neural network algorithm, and fast and accurate automated classification for massive image datasets using the Hadoop platform. The completed implementation was tested with image data from the Pascal VOC2007, Caltech256 and SUN datasets. The experimental results verify that the proposed algorithm not only can handle large-scale datasets but also scales very well in terms of the evaluation metrics of speedup, sizeup and scaleup. The experimental results show that the algorithm proposed in this study achieves good parallelization that can make full use of distributed system resources to improve the algorithm’s classification performance. In addition, the distributed parallel system based on MapReduce greatly improved performance relative to traditional single node algorithm architectures and fully demonstrates the powerful computing ability of parallel processing. Because the parallel design and implementation of algorithms is a new and hot research topic, in our future work, we intend to explore designing optimized Map and Reduce tasks for the MapReduce parallel programming model.

## Methods

### The MapReduce parallel programming model

**Figure 9:**
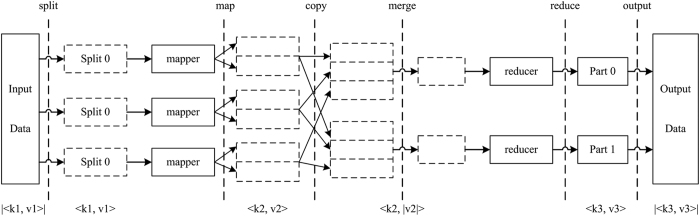
MapReduce implementation process.

MapReduce is a core technology of distributed parallel processing on the Hadoop platform that uses a standard functional programming calculation model and divides the calculation into two tasks, Map and Reduce, which correspond to the mapper() and reducer() functions, respectively^32^. The two functions use key/value pairs and convert the input key-value pair (<*key*_*i,*_*value*_*i*_>) to an output key-value pair (<*key*_*j,*_*value*_*j*_>) according to certain mapping rules. The goal of the Map task is to decompose a large dataset into a set of smaller datasets (*split*) and then calculate and generate intermediate results using free nodes in the Hadoop cluster. The Reduce task traverses and sorts the intermediate results generated by the Map task based on specified instructions. Then, it generates the final results. In other words, the MapReduce parallel programming model uses the mapper() function to segment a large dataset (the smaller datasets after segmentation are handled by each computational node), and the Reduce task uses the reducer() function to combine the process results of each node. Together, these functions achieve distributed parallel processing. The MapReduce implementation process is shown in Fig. 9.

### Algorithm design

#### The traditional Adaboost-BP neural network algorithm

To construct a good network structure, the traditional BP neural network algorithm tests the network continually based on experience and repeated experiments to obtain good generalization ability. Hansen and Salamon have proven that repeatedly training multiple neural networks and combining the outputs can significantly improve the generalization ability of neural network algorithms^33^. The AdaBoost algorithm, proposed by Freund and Schapire in 1999, is an iterative algorithm that obtains a sample weight by repeatedly searching the sample characteristic space^30^. It constantly adjusts the weights of training samples during the iterative process (increasing sample weights that have low forecast precision and reducing sample weights that have high forecast precision) and forms a strong classifier by linearly combining learning algorithms to improve their classification performance. The Adaboost algorithm is widely used in all types of practical problems because it does not need to know the lower limits of the weak classification learning algorithms in advance. The Adaboost-BP neural network algorithm is a combination of the Adaboost algorithm and the BP neural network algorithm that uses BP neural networks as the weak classifiers and constructs a strong classifier by integrating the outputs of multiple BP neural networks. The steps of the algorithm are as follows:^34^

1. Initialize the distribution weights of the sample data and the BP neural network. The distribution weights *D*_*t (i)*_ of the sample data are calculated by the formula 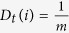, where *m* is the number of training samples. Determine the structure of the BP neural network. The number of nodes in the input layer is determined according to the features of the sample, the number of nodes in the output layer is determined according to the output result dimension, and the number of nodes in the hidden layer is determined by the following formula:
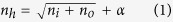
where *n*_*i*_, *n*_*o*_, *n*_*h*_ are the number of nodes of the input, output and hidden layers, respectively, and α is a random number between 0 and 1. The initial weights and threshold values of the BP neural network are initialized to a random number between 0 and 1.
2. Make a predictor of a single BP neural network as a weak classifier. Train the BP neural network and calculate the sum *ε*_*t*_ of its prediction error for the prediction sequence *g(t*):
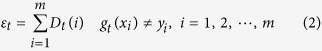
where *g*_*t*_ (*x*_*i*_) is the prediction value of BP neural network and *y*_*i*_ is the desired value.
3. Calculate the weight of the predicted sequence *α*_*t*_ as follows:
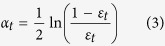
4. Adjust the weights of the samples:
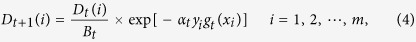
where *B*_*t*_ is a normalization factor whose purpose is to keep the weight sum to 1 for a constant weight proportion.
5. Construct a strong classification function. The strong classification function *h*(*x*) is obtained by integrating *T* groups of weak classification functions *f* (*g*_*t,*_*α*_*t*_) after *T* rounds of training.

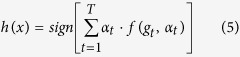

#### Parallel Adaboost-BP neural network algorithm

**Figure 10:**
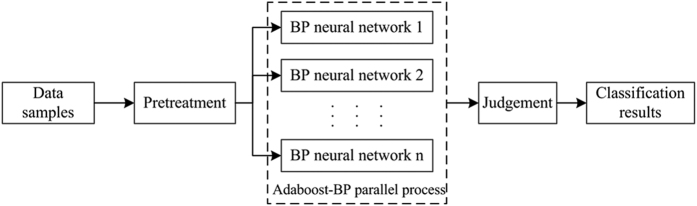
The parallel model of the proposed algorithm.

To overcome the defects of hardware overhead and long training time exhibited by the traditional Adaboost-BP neural network algorithm when processing massive data, this study parallelizes the Adaboost-BP neural network algorithm using the MapReduce parallel programming model, which effectively shortens the training time and improves prediction accuracy. The parallel model is shown in Fig. 10.

1. Design and realization of the Adaboost-BP-mapper() function In the Map stage, aiming at each BP neural network, the mapper() function calculates the output of the network layer by layer, compares the outputs with the desired values, obtains the prediction error *ε*_*t*_, updates the connection weights, and passes the results to the Reduce task. The pseudo-code is as follows:
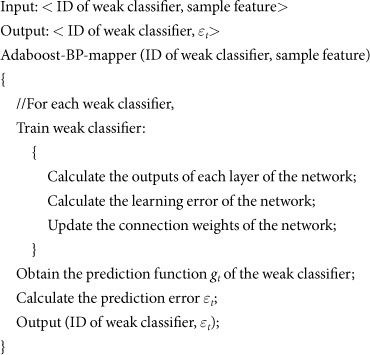
2. Design and realization of Adaboost-BP-combine() function For the MapReduce parallel programming model, a combine() function can be used to perform local processing of the intermediate results generated in the Map stage. This function greatly reduces communication overhead. Therefore, this study designs an Adaboost-BP-combine() function to process the intermediate results produced by the Adaboost-BP-mapper() function locally before entering the Reduce stage. The pseudocode is as follows:
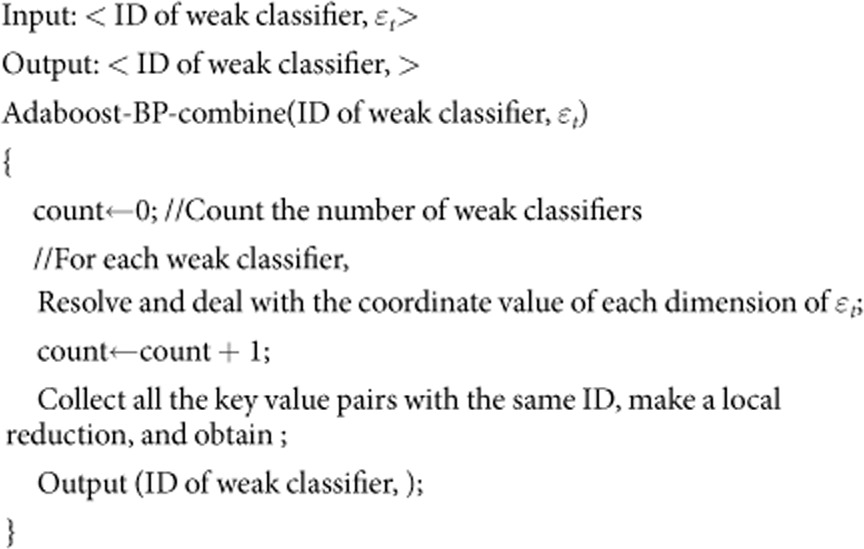
3. Design and implementation of Adaboost-BP-reducer() At the Reduce stage, the reducer() function receives the outputs of the combine() function. It combines and calculates using these values and returns the final outputs. The pseudocode is as follows:

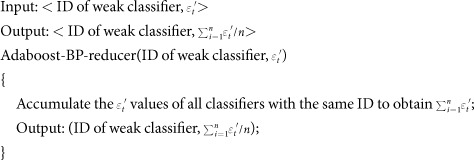


### A massive image dataset classification approach using parallel Adaboost-BP neural network

#### Parallel extraction of image features

Scale-invariant feature transform (SIFT) is an image feature descriptor based on scale space proposed by David G^35^. SIFT is a calculation method that works by detecting pyramid extreme points of multiresolution images to extract the invariant key points of image position, scale and rotation. It has become a widely used image feature extraction algorithm because of its invariance to image scaling, rotation and affine transformation as well as its strong distinguishing capabilities. The calculation process mainly involves constructing a scale space, detecting extreme points and obtaining the scale invariance, filtering and precisely positioning the feature points, distributing the direction value for each feature point, and generating feature descriptors.

After image feature extraction using the SIFT algorithm, a feature matrix of *n* × 128 is generated, in which *n* is the number of key points for each image and 128 represents the feature dimension of each key point. However, different images have different numbers of key points because the number of key points for each image is highly dependent upon the gradient of the image itself. Moreover, *n* is generally the number of key points of the image with the maximum number of key points extracted by SIFT, which causes the generated feature matrix to be a sparse matrix with a large number of zero elements and high redundancy. Therefore, this study improves the classical SIFT algorithm by clustering the feature matrix of *n*×128 to decrease the number of key points when parallelizing the SIFT algorithm based on the MapReduce model. The steps for parallel SIFT feature extraction are as follows.


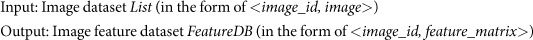


Step 1. Decompose the image dataset *List*. Each image is regarded as a *Split*.

Step 2. The mapper() function receives the input and extracts the features of each image in parallel according to the SIFT algorithm described in and, finally, generates the feature matrix^35^.

Step 3. For the feature matrix in Step 2, the function mapper() clusters the *n* key points using fuzzy C-means (FCM) following^36^. (The number of cluster centers in this study is limited to 100.)

Step 4. The reducer() function receives the outputs of the mapper() function. It collects and merges the data, calculates the means of the key points belonging to the same category in the clustering results, and generates the 100 × 128 feature matrix as the final output.

#### Constructing the classification model

After extracting the SIFT features of images, the classification model for massive image datasets is designed according to the proposed parallel Adaboost-BP neural network algorithm in this study (Fig. 1).

The classification model is based on a Hadoop distributed architecture. First, the model distributes the work. This requires a complex calculation process (e.g., image feature extraction, training the classification model, and predicting classifications) that is distributed among the node computers in the large-scale computer cluster. Second, all node computers execute tasks in parallel, and the master node computer summarizes and merges the intermediate results. Finally, the classification results are fed back to the user.

### Implementing massive image dataset classification

The massive image dataset classification model based on the parallel Adaboost-BP neural network uses the MapReduce parallel programming model from the Hadoop platform to achieve parallel distributed processing for automated massive image dataset classifications. The specific steps are as follows.

1. Extract image features according to the above parallel SIFT feature extraction method. Extract and cluster SIFT features of images in parallel and generate the feature matrix.
2. Determine the topological structure of the Adaboost-BP neural network model according to the previously described traditional Adaboost-BP neural network algorithm. This study used 15 BP neural networks as weak classifiers. The number of weak classifiers is determined by practical experience.
3. Determine the learning sample and normalize the sample data according to Formula (6):
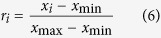
where *x*_1_ is the feature value extracted using the improved SIFT algorithm for the sample, *x*_max_, *x*_min_ are the maximum and minimum of feature values for all sample data, respectively, and *r*_*i*_ is the feature value after normalization.
4. Train the Adaboost-BP neural network in parallel. To train the parallel Adaboost-BP algorithm proposed in this study, build the Hadoop cluster, update the connection weights continuously, correct the errors repeatedly, and combine the outputs of the networks.
5. Make classification predictions. Predict the image category using the trained network structure, and return the results to users.

### Experimental environment and data

The experimental environment for this study consisted of one Hadoop cluster with five computers in an intranet. One computer was designated as the master node and the other four were the slave nodes. All the node computers were equipped with 4 GB quad-core processors, 1 TB hard disks, and the Ubuntu operating system.

The experimental data in this study stemmed from the Pascal VOC2007, Caltech256 and SUN datasets. These three datasets incorporate a variety of categories such as vehicles, animals and plants, and indoor and outdoor scenes. At present, these three large image datasets have been made available for free. The Pascal VOC2007 dataset contains 9,963 images in 20 categories (such as plane, bike, bird, car, and table). The Caltech256 dataset contains 30,607 images in 256 categories (such as basketball, bat, canoe, French horn, and ladder), each of which contains at least 80 images. The SUN dataset contains 131,067 images in 908 categories (such as kitchen, mesa, plaza, wave, and roof garden). In this study, we randomly selected 300 images and 65 images from each category in the Pascal VOC2007 and Caltech256 datasets, respectively, for the training set, and the remaining images were included in the test sets. Because the SUN dataset is a scene image dataset and each category contains different numbers of images, this paper randomly selected 30,000 images (18,000 images for the training set and 12,000 images for the test set) from the dataset for the experiments. For processing convenience, we formatted all the experimental images to a consistent size: 256*256 pixels. To verify the classification performance, experiments are performed to compare the following three aspects: classification accuracy, running time, speedup, sizeup and scaleup.

## Additional Information

**How to cite this article**: Cao, J. *et al*. A Parallel Adaboost-Backpropagation Neural Network for Massive Image Dataset Classification. *Sci. Rep.* **6**, 38201; doi: 10.1038/srep38201 (2016).

**Publisher's note:** Springer Nature remains neutral with regard to jurisdictional claims in published maps and institutional affiliations.

## Figures and Tables

**Table 1:** Classification accuracy (%) of different approaches based on the Pascal VOC2007 dataset.

Image category	RF	SVM	Adaboost-BP^32^	The method of Shi *et al*.^10^.	Parallel BP^29^	The proposed approach
plane	81.9	82.5	82.6	87.2	90.0	94.1
bike	80.0	79.9	79.8	83.3	86.2	90.5
bird	78.2	78.4	78.4	83.9	87.1	92.6
boat	79.9	80.1	80.5	85.0	87.6	94.3
btl	55.0	54.2	54.6	57.1	63.2	73.6
bus	72.8	73.2	73.1	79.6	82.9	87.3
car	81.5	80.8	81.9	86.4	89.8	95.7
cat	83.1	83.2	83.6	86.8	89.4	94.5
chair	65.4	64.9	65.8	70.5	72.6	83.6
cow	66.2	66.5	67.0	71.4	74.0	86.3
table	60.1	60.0	60.3	65.2	70.5	81.6
dog	84.3	84.1	84.7	88.4	90.4	96.5
horse	81.9	82.1	82.4	87.3	91.3	95.9
moto	79.0	79.2	79.3	83.8	87.2	92.8
pers	86.5	86.9	87.3	93.5	95.7	97.1
plant	58.3	59.1	60.2	66.1	71.9	78.6
sheep	73.6	74.1	74.8	79.9	82.6	90.0
sofa	63.5	63.9	65.7	68.3	73.8	79.9
train	81.0	81.4	81.7	85.9	89.1	94.7
TV	73.3	73.9	74.8	78.4	84.2	89.1
Mean	74.3	74.4	74.9	79.4	83.0	89.4
Standard deviation	9.314	9.377	9.213	9.400	8.576	6.634

**Table 2:** Average accuracy (%) of different approaches based on the Caltech256 dataset and SUN dataset.

Image dataset	Classification approach	Min (%)	Max (%)	Average accuracy (%)
Caltech256 dataset	RF	51.7	68.3	59.8
	SVM	51.4	70.6	60.1
	Adaboost-BP^32^	52.9	71.1	60.3
	The method of Shi *et al*.^10^.	60.8	79.5	68.7
	Parallel BP^29^	72.3	86.1	78.7
	The proposed approach	82.2	95.9	86.3
SUN dataset	RF	54.2	70.0	61.9
	SVM	53.7	71.6	62.1
	Adaboost-BP^32^	55.1	71.9	63.4
	The method of Shi *et al*.^10^.	63.3	81.7	72.5
	Parallel BP^29^	73.6	90.5	82.1
	The proposed approach	83.9	96.1	89.2

**Table 3:** Average accuracy (%) of different approaches based on different numbers of images and image categories.

Image categories	Classification approach	Number of images					
		500	1,000	2,000	5,000	10,000	20,000
5	RF	96.1	95.0	91.9	85.8	77.8	67.9
	SVM	96.3	95.1	92.2	86.5	78.6	69.3
	Adaboost-BP	96.3	95.0	92.6	87.4	80.1	71.7
	The method of Shi *et al*.	96.3	95.3	93.5	90.0	85.5	78.3
	Parallel BP	100.0	99.6	99.4	99.2	97.5	94.6
	Parallel Adaboost-BP	100.0	100.0	99.9	99.8	98.0	97.1
10	RF	95.0	93.5	88.9	82.6	74.3	63.8
	SVM	95.1	93.9	89.7	83.8	76.1	65.2
	Adaboost-BP	95.1	94.2	91.4	85.5	77.2	67.0
	The method of Shi *et al*.	95.5	94.7	92.6	89.3	84.5	78.0
	Parallel BP	99.7	99.5	99.3	99.1	96.8	94.0
	Parallel Adaboost-BP	100.0	100.0	99.8	99.7	97.3	96.3
20	RF	93.1	92.0	85.9	77.9	69.1	58.8
	SVM	93.5	92.0	86.7	79.3	70.4	60.5
	Adaboost-BP	94.0	92.1	88.3	81.9	72.6	62.0
	The method of Shi *et al*.	94.0	94.0	91.6	87.2	80.5	71.8
	Parallel BP	99.5	99.4	99.0	97.3	94.1	90.8
	Parallel Adaboost-BP	99.8	99.6	99.5	99.0	97.1	95.3
50	RF	92.7	89.4	82.8	75.0	66.6	56.8
	SVM	92.8	90.1	83.9	76.9	68.1	58.3
	Adaboost-BP	92.8	90.3	85.6	79.1	70.7	60.6
	The method of Shi *et al*.	93.3	93.0	91.0	86.6	78.5	68.9
	Parallel BP	98.8	98.7	98.0	97.0	93.8	89.6
	Parallel Adaboost-BP	99.7	99.0	98.5	97.4	96.0	93.4
100	RF	89.7	87.6	80.1	73.7	64.1	54.1
	SVM	90.3	88.0	81.9	74.2	65.8	56.0
	Adaboost-BP	90.9	88.4	83.7	77.5	68.4	58.3
	The method of Shi *et al*.	92.3	92.1	89.0	83.2	69.7	59.4
	Parallel BP	98.9	97.6	95.0	93.9	90.2	83.1
	Parallel Adaboost-BP	99.5	99.0	98.1	94.8	92.3	90.0
200	RF	87.0	83.6	76.9	68.8	58.1	46.0
	SVM	87.1	84.3	77.8	70.0	60.2	49.2
	Adaboost-BP	87.2	85.1	79.5	72.3	62.4	51.0
	The method of Shi *et al*.	90.3	89.5	87.6	81.2	68.7	58.3
	Parallel BP	98.5	97.1	93.9	90.7	85.3	78.2
	Parallel Adaboost-BP	99.5	98.5	98.1	93.4	90.7	86.1

**Table 4:** The accuracy declines (%) of different approaches as the number of images in each category changes from 5 to 200.

Classification approaches	500 images	1,000 images	2,000 images	5,000 images	10,000 images	20,000 images
RF	9.1	11.4	15.0	17.0	19.7	21.9
SVM	9.2	10.8	14.4	16.8	18.4	20.1
Adaboost-BP	9.1	9.9	13.1	15.1	17.7	20.7
The method of Shi *et al*.	6.0	5.8	5.9	8.8	16.8	20.0
Parallel BP	1.5	2.5	5.5	8.5	12.2	16.4
Parallel Adaboost-BP	0.5	1.5	1.8	6.4	7.3	11.0

**Table 5:** The number of correctly classified images and incorrectly classified images in the test sets of the three datasets.

Classification approaches	The number of correctly classified images	The number of incorrectly classified images
RF	18,725	11,205
SVM	18,795	11,135
Adaboost-BP	18,998	10,932
The method of Shi *et al*.	21,442	8,488
Parallel BP	24,133	5,797
Parallel Adaboost-BP	26,300	3,630

**Table 6:** Running times for the different approaches.

(a) Training time (s)				
Classification approach	Image category	1,000 images	5,000 images	15,000 images
RF	10	56	373	5,874
	30	58	388	6,137
	100	67	404	6,581
SVM	10	55	371	5,869
	30	58	386	6,130
	100	68	402	6,573
Adaboost-BP	10	56	372	5,872
	30	59	388	6,135
	100	67	403	6,579
The method of Shi *et al*.	10	55	370	5,431
	30	59	391	5,955
	100	68	401	6,417
Parallel BP	10	12	47	139
	30	14	50	149
	100	19	54	155
Parallel Adaboost-BP	10	11	45	129
	30	14	49	133
	100	18	52	148
**(b) Testing time (ms)**				
RF	10	4	6	10
	30	4	7	13
	100	6	11	16
SVM	10	4	6	9
	30	4	7	12
	100	6	10	15
Adaboost-BP	10	4	6	9
	30	4	7	12
	100	6	10	16
The method of Shi *et al*.	10	4	5	9
	30	5	7	11
	100	5	10	14
Parallel BP	10	1	2	4
	30	1	2	4
	100	1	3	4
Parallel Adaboost-BP	10	1	2	3
	30	1	2	3
	100	1	2	4
